# Enhanced architecture and implementation of spectrum shaping codes

**DOI:** 10.7717/peerj-cs.1883

**Published:** 2024-02-21

**Authors:** Bingrui Wang, Zhaopeng Xie, Xingang Zhang

**Affiliations:** 1 Henan Engineering Research Center of Service and Guarantee for Intelligent Emergency, Nanyang Normal University, Nanyang, Henan, China; 2 The School of Advanced Manufacturing, Fuzhou University, Jinjiang, Fujian, China

**Keywords:** Spectrum shaping, K-constraint, Guided scrambling, Accumulated signal power, Spectrum null

## Abstract

Spectral shaping codes are modulation codes widely used in communication and data storage systems. This research enhances the algorithms employed in constructing spectral shaping codes for hardware implementation. We present a parallel scrambling calculation with a time complexity of *O*(1). Second, in the minimum accumulated signal power (MASP) module, the sine-cosine accumulation needs to be determined by remainder with time complexity *O*(*n*^2^). We offer reduced MASP computations for short bit-width data, ROM storage, and addition pipelines. It can remove the remainder operation, reducing accumulated complexity to *O*(1). In addition, we present a search algorithm to generate segmented lines to replace the square operations in the MASP module. By employing the search algorithm and shift operations, we can reduce the complexity of the square from *O*(*n*^2^) to *O*(1). The implementation results reveal that the original and proposed MASPs yield nearly identical spectrum nulls. The encoder-decoder of the spectral shaping codes with proposed approaches consumes just 6% of the hardware resources when carried out with a Spartan6 XC6SLX25.

## Introduction

Spectrum shaping codes are categorized as modulation codes, and they are applied initially to digital communications utilizing transformers to connect two lines. Transformers cannot transfer signals without significant distortion if the power spectral densities of signals include low frequency components. The shaping codes are designed to adjust source data to satisfy the features of the communication channel. These codes are also employed for digital recording systems to translate an arbitrary data sequence into a sequence with particular characteristics required by the systems (Immink & Cai, 2021). More recently, a novel concept of integrated microwave photonics spectral shaping is introduced to open avenues to advanced functionalities (Daulay et al., 2021).

Spectrum shaping technologies are utilized in a variety of fields. (1) In information processing and transmission fields, Chai et al. (2014) discuss the practical obstacles to implementing dynamic spectrum access (DSA) devices and offer solutions. In order to accommodate DSA in commercial off-the-shelf wireless devices, they also propose a general per-frame spectrum-shaping protocol. A simple spectrum shaping technique based on switching three loads has been presented for backscatter modulation-based Internet of Things (IoT) systems (Nagaraj, 2017). Danila (2021) describes theoretical research conducted in the terahertz G-band for a piezoelectrically-responsive ring-cone element metasurface composed of polyvinylidene fluoride (PVDF)/silicon and PVDF/silica glass. Utilizing the longitudinal piezoelectric effect of PVDF, this study examines the spectrum shaping ability of a polymer-based metasurface. Three distinct filter functions, such as Fano-like resonances, wavelength interleaving, and variable resonance mode splitting, are accomplished in Arianfard et al. (2021). The outcomes theoretically validate the proposed device as a compact photonic filter with many functions for adjustable spectral shaping. Dobre et al. (2021) developed spectrum-skirt-filled pulse-shaping filters corresponding to spectral mask response. The suggested system design achieves more excellent data rates in a dispersive microwave propagation environment than conventional transmission using Nyquist pulse shaping. Nasarre et al. (2021) present a novel concept of frequency-domain spectral shaping (FDSS) with spectral extension for the enhancement of the uplink (UL) coverage in 5G New Radio (NR), based on discrete Fourier transform spread orthogonal frequency-domain multiplexing. The results demonstrate that the spectrally-extended FDSS method is a highly effective solution for improving the 5G NR UL coverage. Furthermore, we can create dependable systems by integrating modern modulation techniques and rate-diverse error-correcting codes (Fang et al., 2023; Chen et al., 2023; Lin et al., 2023). (2) In information storage fields, spectrum shaping codes have spectrum nulls at specific frequencies (Pelusi et al., 2015). In addition, it is expected to enhance the performance of dedicated servo recording systems by using the shaping codes (Ng et al., 2015; Yuan et al., 2015), which is a promising technology for ultra-mobile hard disk drives. Shaping codes with spectrum nulls at non-zero frequencies effectively reduces interference between data signals and narrow band signals. In a dedicated servo recording system, there are two frequencies for servo signals, *i.e.,* a frequency of *f*_1_ on even tracks and a frequency of *f*_2_ on odd tracks. In addition to avoiding interference between data and servo signals, it also permits filtering of low-frequency disc noise. Moreover, the applied recording systems require a run-length limit constraint (Tandon, Motani & Varshney, 2019), also known as the *k*-constraint. Kahlman & Immink (1995) concern the spectral shaping of both embedded pilot tones and spectral nulls in digital magnetic video tape recordings. The spectral notches are essential to prevent interference between the written data and the servo detecting mechanism. (3) In medical fields, Greffier et al. (2020) investigate the influence of tin filter-based spectral shaping computed tomography (CT) on image quality and radiation dose for use in ultralow-dose CT protocols. Tin filtering enhances the quality of the X-ray beam and the image quality characteristics of phantom images. Baldi et al. (2020) suggest a spectral shaping and third-generation dual-source multidetector CT scanner for evaluating osteolytic lesions caused by multiple myeloma. The outcome validates the benefits of whole-body low dose computed tomography for diagnosing patients with multiple myeloma. Agostini et al. (2021) investigate the function of third-generation iterative reconstruction (ADMIRE3) in a dual-source, high-pitch chest CT protocol with spectral shaping at 100 kVp coronavirus disease 2019 (COVID-19). The low-dose CT with spectral shaping and ADMIRE3 provides acceptable image quality for evaluating COVID-19 patients while significantly reducing radiation dose and motion anomalies. Hardening the X-ray beam, tin prefiltration is established for imaging high-contrast subjects in energy-integrating detector computed tomography (EID-CT) (Grunz et al., 2022). This study aims to examine the potential dose-saving effect of spectral shaping *via* tin prefiltration in photon-counting detector CT (PCD-CT) of the temporal bone. Seeking for matched image noise, high-voltage scan methods with tin prefiltration enables more significant dose savings in EID-CT. However, superior inherent denoising reduces the dose reduction potential of spectral shaping in PCD-CT.

Based on the excellent performance of the research in Cai et al. (2017), this study presents the implementation of spectrum shaping codes deploying a field programmable gate array (FPGA). The shaping code architecture consists of scrambling and descrambling, *k*-constrained encoding and decoding (Immink, 2012), and a minimum accumulated signal power (MASP) module. We provide simplified approaches for these modules, which are suitable for hardware implementation. Scrambling is a highly effective technique (Park & Son, 2020; Xiao et al., 2020; Liu et al., 2021). In the proposed scrambling and descrambling, we use only XOR (exclusive or) logical operations and no other arithmetic operations. The algorithm for *k*-constrained encoding and decoding is then described. Furthermore, we propose improved calculations to reduce parameter storage and processing complexity in the MASP module.

The study is organized as follows. In ‘Shaping Code Algorithms’, we describe the overall architecture of the FPGA system implementation and present the algorithms of spectrum shaping codes. ‘The enhanced algorithms’ enhances the algorithms. ‘FPGA implementation of a spectrum shaping code’ demonstrates a specific hardware implementation of the shaping algorithms with reduced computations. The shaping code is synthesized, and the consumed resources are analyzed. ‘Discussion and conclusion’ gives the conclusion and discussion.

## Shaping Code Algorithms

**Figure 1:**
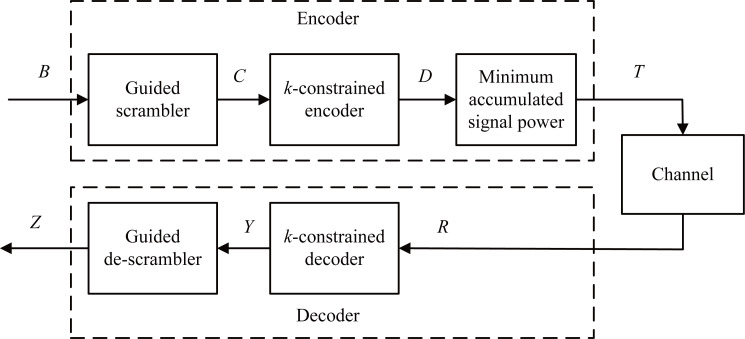
Schematic diagram of the spectrum shaping code with guided scrambling.

Figure 1 illustrates a block diagram of an encoder and a decoder for spectrum shaping codes. In the encoder, the first step is to generate 0 to 2^*p*^ − 1 numbers in decimal form and convert them to binary vector *A* with the size of 2^*p*^ × *p*, and *p* denotes the length of a scrambler. Then, we append *A* to the user data of length *m* bits, generating a vector *B* of size 2^*p*^ × (*m* + *p*), that is, *B* = [*B*(0), *B*(1), …, *B*(2^*p*^ − 1)]. Second, *B* is fed into a guided scrambler module and then is scrambled. Then, we can generate a vector *C* of size 2^*p*^ × (*m* + *p*). Third, the scrambled vector *C* is encoded using a *k*-constrained encoder, yielding a vector *D* of size 2^*p*^ × (*m* + *p* + 1). Finally, the accumulated signal power is calculated from *D*(0) to *D*(2^*p*^ − 1), and the one with the least power vector *T* is chosen and sent. In the decoding process, the received signal *R* with a bit-width (*m* + *p* + 1) is fed into the *k*-constrained decoder, which produces the data *Y* with a bit-width (*m* + *p*). By descrambling *Y*, we can obtain the data *Z* with a bit-width (*m* + *p*). The original user data can be recovered by eliminating the redundant *p* bits.

### Simplified scrambling and unscrambling algorithms

In this study, the guided scrambler (GS) polynomial is

(1) \begin{eqnarray*}g\mathrm{(}x\mathrm{) = }{g}_{0}+{g}_{1}x+\cdots +{g}_{p-2}{x}^{p-2}+{g}_{p-1}{x}^{p-1}+{x}^{p}.\end{eqnarray*}

Where *g*_0_ is a constant bit of value 1, and *g*_*i*_ is binary bit of 1 or 0, 0 < *i* < *p*. The $ \left\{ {b}_{0},{b}_{1},{b}_{2},\ldots ,{b}_{n-2} \right\} $ is the bit set to be scrambled. The $ \left\{ {c}_{0},{c}_{1},{c}_{2},\ldots ,{c}_{n-2} \right\} $ represents the scrambled bit set. The *b*_*i*_ and *c*_*i*_ are the binary bits, 0 ≤ *i* ≤ *n* − 2. Each value of $ \left\{ {c}_{0},{c}_{1},\ldots ,{c}_{p-1} \right\} $ is initialized to zero. The bits of $ \left\{ {c}_{p},{c}_{p+1},\ldots ,{c}_{n-2} \right\} $ can be generated by employing the encoding as

(2) \begin{eqnarray*}{c}_{i}\mathrm{ = }{b}_{i}+\sum _{k=0}^{p-1}{g}_{p-k-1}{c}_{i-k-1},p\leq i\leq n-2.\end{eqnarray*}

As *b*_*i*_, *c*_*i*_, and *g*_*j*_ are binary values of 1 or 0, 0 ≤ *i* ≤ *n* − 2, 0 ≤ *j* < *p*, and then the multiplication result of *g*_*i*−*k*−1_*c*_*i*−*k*−1_ is also binary. Moreover, logical XOR operation can replace the addition involved in Eq. (2). The XOR compares two bits and returns a bit 1 if the two bits are different, 0 if they are equal (Qiu et al., 2024). An XOR operation between a variable and 0 returns the variable itself. Let the operation ⊕ indicate bitwise XOR. Therefore, we can modify Eq. (2) as

(3) \begin{eqnarray*}{c}_{i}\mathrm{ = }{g}_{0}{c}_{i-p}\oplus {g}_{1}{c}_{i-p+1}\oplus \cdots \oplus {g}_{p-1}{c}_{i-1}\oplus {b}_{i},p\leq i\leq n-2.\end{eqnarray*}

Since Eq. (3) has a recursive structure, we perform a serial implementation, which takes *n* clock cycles to complete. Thus, the time complexity of GS encoding is *O*(*n*).

Next, we show the GS decoding process for restoring the original bit set $ \left\{ {b}_{0},{b}_{1},{b}_{2},\ldots ,{b}_{n-2} \right\} $ from the encoded bit set $ \left\{ {c}_{0},{c}_{1},{c}_{2},\ldots ,{c}_{n-2} \right\} $. The GS encoding and decoding involve only XOR operations. In order to get *b*_*i*_ from *c*_*i*_, we use Equation (2) or (3) and add *b*_*i*_⊕*c*_*i*_ on the both hands side of Eqs. (2) or (3). Hence, we get the desired last Eq. (4) generating the values of *b*_*i*_.

(4) \begin{eqnarray*}\begin{array}{@{}l@{}} \displaystyle {b}_{i}={b}_{i}\oplus \sum _{k=0}^{p-1}{g}_{p-k-1}{c}_{i-k-1}\oplus {b}_{i}\oplus {c}_{i}\\ \displaystyle ={c}_{i}\oplus \sum _{k=0}^{p-1}{g}_{p-k-1}{c}_{i-k-1}\mathrm{,}p\leq i\leq n-2. \end{array}\end{eqnarray*}

By comparing Eqs. (2) with (4), we observe that we only need to change positions between *c*_*i*_ and *b*_*i*_. Since the *c*_*i*_ is the encoded bit and can be known, the GS decoding can be implemented in parallel.

### The algorithm of *k*-constrained encoding and decoding

The *k*-constrained encoding algorithm is described as follows:

Step 1: Add a bit 1 to the scrambled bit set $c= \left\{ {c}_{0},{c}_{1},\ldots ,{c}_{n-2} \right\} $, and then generate $e= \left\{ {e}_{0},\ldots ,{e}_{n-1} \right\} $, where *e*_0_ = 1 and (*c*_0_, *c*_1_, …, *c*_*n*−2_) = (*e*_1_, *e*_2_, …, *e*_*n*−1_).

Step 2: The bit set *e* is splitted into *L* blocks of *q* bits, where *L* = 2^*q*−1^ and *n* = *L*∗*q*. The *L* blocks consist of *L*_0_ = (*e*_0_, *e*_1_, …, *e*_*q*−1_), *L*_1_ = (*e*_*q*_, *e*_*q*+1_, …, *e*_2*q*−1_), ⋯, *L*_*i*_ = (*e*_*i*∗*q*_, *e*_*i*∗*q*+1_, …, *e*_(*i*+1)∗*q*−1_), ⋯, *L*_2^*q*^^−1^−1_ = (*e*_*n*−*q*_, *e*_*n*−*q*+1_, …, *e*_*n*−1_), 0 ≤ *i* ≤ (2^*q*^^−1^ − 1).

Step 3: If (*e*_*i*∗*q*_, *e*_*i*∗*q*+1_, …, *e*_(*i*+1)∗*q*−1_) = [0]_*bin*_, then (*e*_*i*∗*q*_, *e*_*i*∗*q*+1_, …, *e*_(*i*+1)∗*q*−1_) = (*e*_*u*∗*q*_, *e*_*u*∗*q*+1_, …, *e*_(*u*+1)∗*q*−1_), where 0 ≤ *u* ≤ *i* ≤ (2^*q*^^−1^ − 1) and the []_*bin*_ represents to take a binary number with *q* bits.

Step 4: (*e*_*u*∗*q*_, *e*_*u*∗*q*+1_, …, *e*_(*u*+1)∗*q*−1_) = [*i*]_*bin*_. Repeat steps 3 and 4 *L* − 1 times.

Step 5: If (*e*_*i*∗*q*_, *e*_*i*∗*q*+1_, …, *e*_(*i*+1)∗*q*−1_) = [2^*q*^ − 1]_*bin*_ and the first bit of (*e*_*u*∗*q*_, *e*_*u*∗*q*+1_, …, *e*_(*u*+1)∗*q*−1_) is 1, then (*e*_*i*∗*q*_, *e*_*i*∗*q*+1_, …, *e*_(*i*+1)∗*q*−1_) = [0]_*bin*_. Repeat step 5 *L* times.

The *k*-constrained decoding algorithm is presented as follows:

Step 1: If (*e*_*i*∗*q*_, *e*_*i*∗*q*+1_, …, *e*_(*i*+1)∗*q*−1_) = [0]_*bin*_, we can have (*e*_*i*∗*q*_, *e*_*i*∗*q*+1_, …, *e*_(*i*+1)∗*q*−1_) = [2^*q*^ − 1]_*bin*_, 0 ≤ *i* ≤ (2^*q*^^−1^ − 1). Repeat step 1 *L* times.

Step 2: If the first bit of (*e*_*i*∗*q*_, *e*_*i*∗*q*+1_, …, *e*_(*i*+1)∗*q*−1_) is 0, we have *z* = [(*e*_*i*∗*q*_, *e*_*i*∗*q*+1_, …, *e*_(*i*+1)∗*q*−1_)]_*dec*_ and (*e*_*z*∗*q*_, *e*_*z*∗*q*+1_, …, *e*_(*z*+1)∗*q*−1_) = [0]_*bin*_, where the []_*dec*_ represents to take a decimal number. Repeat step 2 *L* times. Note that if the first bit *e*_0_ is 1, the step 2 is executed only once, and the decoded data is obtained by simply removing the first bit *e*_0_.

### The algorithm of minimum accumulated signal power

As given in Cai et al. (2017), the MASP criterion is

(5) \begin{eqnarray*}\sum _{s=1}^{t}{ \left\vert \sum _{i=1}^{n(l-1)}{w}_{i}^{\ast }{e}^{-j2\pi {f}_{s}i}+\sum _{i=(l-1)n+1}^{nl}{w}_{i}{e}^{-j2\pi {f}_{s}i} \right\vert }^{2}.\end{eqnarray*}

Where $j=\sqrt{-1}$, *t* is the number of spectrum nulls at frequencies *f*_1_, *f*_2_, ...,  *f*_*t*_ and *n* is the length of one codeword, *l* indicates the number of codewords that need to be computed. The *w* and *w*^∗^ express the current unencoded codeword and previous encoded codeword, respectively.

Let

(6) \begin{eqnarray*}\begin{array}{@{}l@{}} \displaystyle R{e}_{s}^{l-1}={ \left[ \sum _{i=1}^{n(l-1)}{w}_{i}^{\ast }{e}^{-j2\pi {f}_{s}i} \right] }_{\mathrm{real}\mathrm{part}}=\sum _{i=1}^{n(l-1)}{w}_{i}^{\ast }\cos \nolimits (2\pi {f}_{s}i),\\ \displaystyle I{m}_{s}^{l-1}={ \left[ \sum _{i=1}^{n(l-1)}{w}_{i}^{\ast }{e}^{-j2\pi {f}_{s}i} \right] }_{\mathrm{imaginary}\mathrm{part}}\mathrm{ \times }-1=\sum _{i=1}^{n(l-1)}{w}_{i}^{\ast }\sin \nolimits (2\pi {f}_{s}i), \end{array}\end{eqnarray*}

where $R{e}_{s}^{l-1}$ and $I{m}_{s}^{l-1}$ are the sine-cosine accumulations of the *l* − 1 codewords and have been calculated. In Eq. (5), the left part is already obtained and can be directly added to the right part. By application of Euler’s formula, the unencoded codeword is computed by

(7) \begin{eqnarray*}\begin{array}{@{}l@{}} \displaystyle \sum _{s=1}^{t}{ \left\vert R{e}_{s}^{l-1}-jI{m}_{s}^{l-1}+\sum _{i=(l-1)n+1}^{nl}{w}_{i} \left[ \cos \nolimits (2\pi {f}_{s}i)-j\sin \nolimits (2\pi {f}_{s}i) \right] \right\vert }^{2}\\ \displaystyle =\sum _{s=1}^{t}{ \left\vert R{e}_{s}^{l-1}+\sum _{i=(l-1)n+1}^{nl}{w}_{i}\cos \nolimits (2\pi {f}_{s}i)-j \left[ I{m}_{s}^{l-1}+\sum _{i=(l-1)n+1}^{nl}{w}_{i}\sin \nolimits (2\pi {f}_{s}i) \right] \right\vert }^{2}\\ \displaystyle =\sum _{s=1}^{t} \left\{ { \left[ R{e}_{s}^{l-1}+\sum _{i=(l-1)n+1}^{nl}{w}_{i}\cos \nolimits (2\pi {f}_{s}i) \right] }^{2}+{ \left[ I{m}_{s}^{l-1}+\sum _{i=(l-1)n+1}^{nl}{w}_{i}\sin \nolimits (2\pi {f}_{s}i) \right] }^{2} \right\} . \end{array}\end{eqnarray*}

## The enhanced algorithms

### Parallel scrambling algorithms

The GS scrambler polynomial is employed as 1 + *x*^2^, where *p*, *g*_0_, and *g*_1_ are equivalent to the digits 2, 1, and 0, respectively. Based on the scrambling Eq. (2), the encoding is given by

(8) \begin{eqnarray*}\begin{array}{@{}l@{}} \displaystyle {c}_{i}\mathrm{ = }{c}_{i-2}\oplus {b}_{i},2\leq i\leq n, \end{array}\end{eqnarray*}

where *c*_0_ and *c*_1_ are initially set to zero. We can see that this calculation is recursively executed in serial. In other words, one clock is taken to produce one *c*_*i*_. It takes *n* clocks to calculate all *c*_*i*_. Thus, the time complexity of the initial scrambling polynomial is *O*(*n*). To reduce this time consumption, we transform Eq. (8) as

(9) \begin{eqnarray*}\begin{array}{@{}l@{}} \displaystyle \left\{ \begin{array}{@{}l@{}} \displaystyle {c}_{2j}\mathrm{ = }{c}_{0}\oplus {b}_{2}\oplus \cdots \oplus {b}_{2j},\\ \displaystyle {c}_{2j+1}\mathrm{ = }{c}_{1}\oplus {b}_{3}\oplus \cdots \oplus {b}_{2j+1},1\leq j\leq \frac{n-2}{2} . \end{array} \right. \end{array}\end{eqnarray*}

In this way, the calculation Eq. (9) is not recursive since the right side of Eq. (9), *i.e.,* input data *b* and *c*_0_ and *c*_1_, are known in advance. Then, we can independently compute *c*_3_, *c*_4_, …, *c*_*n*_ at one clock in parallel, with a time complexity of *O*(1). It means that the time complexity is reduced from *O*(*n*) in Eq. (8) to *O*(1).

The corresponding decoding is given by

(10) \begin{eqnarray*}\begin{array}{@{}l@{}} \displaystyle {b}_{i}={c}_{i-2}\oplus {b}_{i}\oplus {c}_{i-2}\mathrm{,}\\ \displaystyle ={c}_{i}\oplus {c}_{i-2},2\leq i\leq n. \end{array}\end{eqnarray*}

Recall that the encoding and decoding of scrambling only use XOR operations.

### Improved MASP with remainder operation

Solving for sine and cosine is a critical step in Eq. (7), and we propose a minimum accumulated signal power with remainder (MASP-R), which is stated as follows.

Step 1: Convert the radian value 2*πf*_*s*_*i* to the degree value *h*_1_, (*l* − 1)*n* + 1 ≤ *i* ≤ *ln*.

Step 2: The *h*_1_ may be greater than 360 degrees, so we need to perform the remainder of the operation on *h*_1_. Furthermore, since *sin*(*h*_1_) = *cos*(*h*_1_ + 270°), we also need to calculate the remainder of *h*_1_ + 270° to 360°. Thus, the sine and cosine of 2*πf*_*s*_*i* can be given by

(11) \begin{eqnarray*} & {h}_{r1}=mod \left( {h}_{1},360\textdegree \right) , & \cos \nolimits (2\pi {f}_{s}i)=\cos \nolimits {h}_{r1},0\textdegree \leq {h}_{r1}\leq 360\textdegree , & {h}_{r2}=mod \left( {h}_{1}+270\textdegree ,{360}^{o} \right) , & \sin \nolimits (2\pi {f}_{s}i)=\sin \nolimits ({h}_{1})=\cos \nolimits {h}_{r2},0\textdegree \leq {h}_{r2}\leq 360\textdegree .\end{eqnarray*}

Step 3: We construct a ROM and store the cosine values from 0 degrees to 359 degrees in the ROM. Determine the cosine values of *h*_*r*1_ and *h*_*r*2_ from the ROM.

Thus, given the cosine value in the first quadrant, we can determine the values in the other three quadrants. The ROM only needs to store 91 numbers from 0 to 90 degrees instead of 360 values, thus saving 3/4 of the storage space. When MASP solves for sine and cosine, it solves for cos(*h*_*r*1_) and cos(*h*_*r*2_). Next, we show an example of a modified cosine solution using *h*_*r*1_.

(12) \begin{eqnarray*}\cos \nolimits \left( h{r}_{1} \right) = \left\{ \begin{array}{@{}l@{}} \displaystyle \cos \nolimits h{r}_{1},0\textdegree \leq h{r}_{1}\leq 90\textdegree ,\\ \displaystyle -\cos \nolimits (180\textdegree -h{r}_{1}),90\textdegree < h{r}_{1}\leq 180\textdegree ,\\ \displaystyle -\cos \nolimits (h{r}_{1}-180\textdegree ),180\textdegree < h{r}_{1}\leq 270\textdegree ,\\ \displaystyle \cos \nolimits (36{0}^{\mathrm{o}}-h{r}_{1}),27{0}^{\mathrm{o}}< h{r}_{1}< 36{0}^{\mathrm{o}}. \end{array} \right. \end{eqnarray*}

### Improved MASP with no remainder and square

#### Remove the remainder operation

Next, we propose an improved MASP algorithm with no remainder and square (MASP-NRS). According to the MASP formula Eq. (7), we compute a 360-degree remainder, obtain the related sine-cosine value, and perform an addition. A sine-cosine accumulation requires *n* clocks. The parallel execution for the accumulation is complex, and serial operation is employed instead. The time complexity of the remainder operation is *O*(*n*), while a sine-cosine accumulation requires *n* remainders. It leads to the time complexity of accumulation *O*(*n*^2^). To reduce this time complexity, we propose an algorithm to remove the remainder operation that includes the following methods.

Improvement 1: Reduce the number of codewords involved in accumulation. The ${\mathop{\sum }\nolimits }_{i=(l-1)n+1}^{nl}{w}_{i}\cos (2\pi {f}_{s}i)$ and ${\mathop{\sum }\nolimits }_{i=(l-1)n+1}^{nl}{w}_{i}\sin (2\pi {f}_{s}i)$ of the current *l*-th codeword need to be added to the ${\mathop{\sum }\nolimits }_{i=1}^{nl}{w}_{i}^{\ast }\cos (2\pi {f}_{s}i)$ and ${\mathop{\sum }\nolimits }_{i=(l-1)n+1}^{nl}{w}_{i}^{\ast }\sin (2\pi {f}_{s}i)$ of the previous (*l* − 1) codewords. We have

(13) \begin{eqnarray*}\begin{array}{@{}l@{}} \displaystyle R{e}_{s}^{l}=\sum _{i=1}^{n(l-1)}{w}_{i}^{\ast }\cos \nolimits (2\pi {f}_{s}i)+\sum _{i=(l-1)n+1}^{nl}{w}_{i}\cos \nolimits (2\pi {f}_{s}i),\\ \displaystyle I{m}_{s}^{l}=\sum _{i=1}^{n(l-1)}{w}_{i}^{\ast }\sin \nolimits (2\pi {f}_{s}i)+\sum _{i=(l-1)n+1}^{nl}{w}_{i}\sin \nolimits (2\pi {f}_{s}i). \end{array}\end{eqnarray*}

The values of $R{e}_{s}^{l}$ and $I{m}_{s}^{l}$ increase as the number of codewords increases. After accumulating 64 codewords, we reset $R{e}_{s}^{l}$ and $I{m}_{s}^{l}$ to 0 to limit these values.

Improvement 2: Eliminate the remainder of the operation and use ROM storage instead. Let the shaping code utilize two dual servo frequencies, *f*_1_ and *f*_2_. A codeword *w* has *n* bits that is multiplied by four groups of sine-cosine *cos*(2*πf*_1_*i*), *sin*(2*πf*_1_*i*), *cos*(2*πf*_2_*i*), *sin*(2*πf*_2_*i*), and 0 ≤ *i* ≤ *n* − 1. Each sine-cosine group contains *n* data points. A total of 64×4×*n* sine-cosine values are stored.

Improvement 3: Adopt small bit-width. The initial sine-cosine values need to be transformed from decimals to integers to calculate on the FPGA. Multiply the initial sine-cosine values by 15 and round to the nearest integer number, which is approximately equivalent to moving the values left by four bits. As a result, including the sign bit, the bit width of four groups of sine-cosine values is 5, with a maximum value of 15.

Improvement 4: The parallel operation. Each item in codeword *w* has a value of either -1 or 1. The select operations can multiply *w* by sine and cosine. We can acquire *n* sine-cosine values from ROM at the same time and perform parallel selection operations to complete the sine-cosine accumulation in a single clock cycle. Thus, we eliminate the remainder operation, reducing the accumulation time complexity from *O*(*n*^2^) to *O*(1).

#### Remove the square operation

Equation (7) involves a square operation, which has a calculating cost of *O*(*n*^2^) and is challenging to compute. We provide a segmented line search algorithm with dynamic error. The search algorithm seeks segmented points, which are combined to produce a segmented curve. Applying the curve, we obtain an approximate estimation. The operation of this curve only involves deterministic shifts and additions/subtractions with a complexity of *O*(1). The complexity of the proposed search algorithm is two orders of magnitude lower than that of the square operation. The key features of the algorithm are the usage of dynamical error and the balanced coefficient of mean square error. The search algorithm is described in Algorithm 1 .


 
_____________________________________________________________________________ 
 
Algorithm 1 Segmented line search algorithm with dynamical error 
Require: f(x): the square function; x: the independent variable; 
Ensure: a set of segmented points; 
  1:  x ∈ [x0, x1, ⋅⋅⋅, xn−1], k=1; 
  2:  xb = x0, xv = x2, sp0 = x0; 
  3:  for j = 2; j <= n − 1; j + +  do 
 4:       while xb < xi < xv do 
 5:            compute  ˆ f (xi) = f(xv)−f(xb) 
    xv−xb    (xi − xb) + f(xb); 
  6:       end while 
 7:       compute error =  v−1 
   ∑ 
 i=b+1       [ ˆ f (xi)−f(xi)]2 
            ____________________________________ 
(v−b−1)e| ˆ f (xi)−f(xi)|α 
       β        ; 
  8:       if error ≥|f(xv) − f(xb)|μ then 
 9:            spk = xv−1; 
10:            xb = xv; 
11:            xv = xv+2; 
12:            k = k + 1; 
13:       else 
14:            xb = xb; 
15:            xv = xj; 
16:       end if 
17:  end for 
18:  return sp = [sp0, sp1, sp2, ...]; 
_____________________________________________________________________________    


We generally use expression Eq. (14) to calculate the mean square error.

(14) \begin{eqnarray*}\begin{array}{@{}l@{}} \displaystyle error= \frac{\sum _{i=1}^{n}{ \left[ \hat {f}({x}_{i})-f({x}_{i}) \right] }^{2}}{n} \end{array}\end{eqnarray*}

where $\hat {f}({x}_{i})$ represents the predicted value and *f*(*x*_*i*_) the actual value.

The large, varied item significantly influences the error expression Eq. (14), but the little various item has a minor impact. So, in Algorithm 1 , we propose a balanced-coefficient mean square error expression Eq. (15) to accurately describe the importance of each item.

(15) \begin{eqnarray*}\begin{array}{@{}l@{}} \displaystyle erro{r}^{\ast }= \frac{\sum _{i=1}^{n}{ \left[ \hat {f}({x}_{i})-f({x}_{i}) \right] }^{2}}{n \left[ {e}^{ \frac{{ \left\vert \hat {f}({x}_{i})-f({x}_{i}) \right\vert }^{\alpha }}{\beta } } \right] } \end{array}\end{eqnarray*}

where *α* and *β* are called fast and slow decay factors, respectively.

Multiple segmented line Eq. (16) can be generated when segmented points are provided. The product of *x* and *k*_0_, *k*_1_, …,  can be replaced by shift operation on *x*. The complexity of calculating *x*2 is *O*(*n*2), whereas applying Eq. (16) and combining with the shift operation to compute the square of *x* decreases the complexity to *O*(1). As a result, we can rewrite Eq. (7) as Eq. (17).

(16) \begin{eqnarray*}\begin{array}{@{}l@{}} \displaystyle \hat {f}(x)= \left\{ \begin{array}{@{}l@{}} \displaystyle {k}_{0}x+{b}_{0},\,s{p}_{0}\leq x\leq s{p}_{1}\\ \displaystyle {k}_{1}x+{b}_{1},\,s{p}_{1}< x\leq s{p}_{2}\\ \displaystyle {k}_{2}x+{b}_{2},\,s{p}_{2}< x\leq s{p}_{3}\\ \displaystyle   \vdots \end{array} \right. \end{array}\end{eqnarray*}

(17) \begin{eqnarray*}\begin{array}{@{}l@{}} \displaystyle \sum _{s=1}^{t} \left\{ \hat {f} \left[ R{e}_{s}^{l-1}+\sum _{i=(l-1)n+1}^{nl}{w}_{i}\cos \nolimits (2\pi {f}_{s}i) \right] +\hat {f} \left[ I{m}_{s}^{l-1}+\sum _{i=(l-1)n+1}^{nl}{w}_{i}\sin \nolimits (2\pi {f}_{s}i) \right] \right\} \end{array}\end{eqnarray*}

The values of the cosine and sine functions range from −1 to 1 in Eq. (17). It can obtain large values of ${ \left[ R{e}_{s}^{l-1}+{\mathop{\sum }\nolimits }_{i=(l-1)n+1}^{nl}{w}_{i}\cos (2\pi {f}_{s}i) \right] }^{2}$ and ${ \left[ I{m}_{s}^{l-1}+{\mathop{\sum }\nolimits }_{i=(l-1)n+1}^{nl}{w}_{i}\sin (2\pi {f}_{s}i) \right] }^{2}$, when the length *n* of a codeword is large and the binary bits *w*_*i*_ are all positive 1. Note that we use the MASP algorithm only for comparison. Thus, we can simultaneously reduce the sum of the two trigonometric functions without affecting the comparison. Then, we can modify Eq. (17) as

(18) \begin{eqnarray*}\begin{array}{@{}l@{}} \displaystyle \sum _{s=1}^{t} \left\{ \hat {f} \left[ \frac{R{e}_{s}^{l-1}+\sum _{i=(l-1)n+1}^{nl}{w}_{i}\cos \nolimits (2\pi {f}_{s}i)}{num} \right] +\hat {f} \left[ \frac{I{m}_{s}^{l-1}+\sum _{i=(l-1)n+1}^{nl}{w}_{i}\sin \nolimits (2\pi {f}_{s}i)}{num} \right] \right\} . \end{array}\end{eqnarray*}

Where *num* expresses an integer.

## FPGA implementation of a spectrum shaping code

Here, we employ a specific shaping code as an example of FPGA implementation. Let the lengths of the shaping code and the original message be 80 and 77 bits. Then we get *L* = 16 and *q* = 80/*L* =5. The GS scrambler polynomial is 1 + *x*^2^, and the length of the scrambler is 2. Two bits are chosen from the binary set 00, 01, 10, 11 and appended to the original message. Next, the 79 bits need to be scrambled utilizing the parallel scrambling algorithms described in ‘Parallel scrambling algorithms’. After that, we add a bit 1 to the scrambled 79 bits to create 80 bits. The *k*-constrained and MASP algorithms are then executed.

### The implementation of MASP-NRS

#### The implementation of removing reminder operation

Let the shaping code utilize two kinds of dual servo frequencies, *f*_1_ = 1/90 and *f*_2_ = 1/60. Each sine-cosine group contains 80 data points. We store 64 × 4× 80 groups of sine-cosine values, requiring a total of 64 × 4 × 80 × 5 = 12.5 KB.

**Figure 2:**
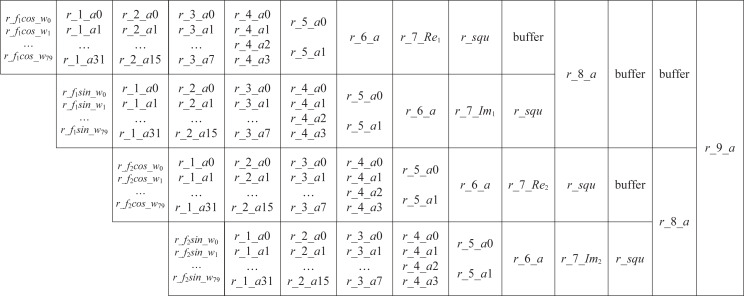
The pipeline of sine-cosine accumulation.

Next, using a pipelined operation, we implement the sine-cosine accumulation in Eq. (18). Figure 2 illustrates the pipeline structure.

Step 1: In Fig. 2, we use *r*_*f*_*k*_*cos*_*w*_*i*_ and *r*_*f*_*k*_*sin*_*w*_*i*_ to denote the product of *w*_*i*_ with *cos*(2*πf*_*k*_*i*) and *sin*(2*πf*_*k*_*i*), *k* = 1, 2, and 0 ≤ *i* ≤ 79. According to the value of *w*_*i*_, we use selectors to determine the 80 values of *r*_*f*_1_*cos*_*w*_*i*_, 0 ≤ *i* ≤ 79.

Step 2: Accumulate *r*_*f*_1_*cos*_*w*_*i*_. If 80 data points are added two by two, the four-stage accumulation will need 40, 20, 10, and 5 addition operations, respectively. Thus, five operands are remaining after the four-stage addition. However, adding these five operands two by two is inconvenient. We construct a segmented accumulation equation because the *r*_*f*_1_*cos*_*w*_*i*_ has a small five-bit width Eq. (19). Applying the equation, the first stage accumulation of 80 data requires only 32 addition operations.

(19) \begin{eqnarray*}\begin{array}{@{}l@{}} \displaystyle \left\{ \begin{array}{@{}l@{}} \displaystyle r\text{_}1\text{_}ai\mathrm{ = }r\text{_}{f}_{1}cos\text{_}{w}_{i\ast 2}+r\text{_}{f}_{1}cos\text{_}{w}_{i\ast 2+1},0\leq i\leq 15,\\ \displaystyle r\text{_}1\text{_}ai\mathrm{ = }r\text{_}{f}_{1}cos\text{_}{w}_{i\ast 2}+r\text{_}{f}_{1}cos\text{_}{w}_{i\ast 2+1}+\\ \displaystyle r\text{_}{f}_{1}cos\text{_}{w}_{i+48},16\leq i\leq 31. \end{array} \right. \end{array}\end{eqnarray*}

Step 3: In Fig. 2, the variables *r*_1_*a*0, …, *r*_1_*a*31, *r*_2_*a*0, …, *r*_2_*a*15, *r*_3_*a*0, …, *r*_3_*a*7, *r*_4_*a*0, …, *r*_4_*a*3, *r*_5_*a*0, …, *r*_5_*a*1, *r*_6_*a*, comprise a six-step sine-cosine cumulative pipeline. A two-by-two addition is then performed, *i.e.,*

(20) \begin{eqnarray*}\begin{array}{@{}l@{}} \displaystyle \left\{ \begin{array}{@{}l@{}} \displaystyle r\text{_}2\text{_}a0=r\text{_}1\text{_}a0+r\text{_}1\text{_}a1,\\ \displaystyle r\text{_}3\text{_}a0=r\text{_}2\text{_}a0+r\text{_}2\text{_}a1,\\ \displaystyle r\text{_}4\text{_}a0=r\text{_}3\text{_}a0+r\text{_}3\text{_}a1,\\ \displaystyle r\text{_}5\text{_}a0=r\text{_}4\text{_}a0+r\text{_}4\text{_}a1. \end{array} \right. ~ \end{array}\end{eqnarray*}

The cumulative result *r*_6_*a* = *r*_5_*a*0 + *r*_5_*a*1. Each *r*_6_*a* of the current codeword needs to be added to the accumulated values of the previous codewords (denoted by *Re*_1_, *Im*_1_, *Re*_2_ and *Im*_2_) to yield *r*_7_*Re*_1_, *r*_7_*Im*_1_, *r*_7_*Re*_2_ and *r*_7_*Im*_2_.

Step 4: Following accumulation, an operation instead of the square is performed, which is introduced in the next section. The accumulation of an encoded codeword is completed after 14 cycles. At the 5th clock, calculate the next encoded codeword. In Fig. 2, the buffer indicates a cache of one clock.

#### The implementation of removing square operation

For Eq. (18), a symbol contains 80 bits. Due to the *k*-constrained algorithm, there will not be five consecutive 1’s and five consecutive 0’s, and a symbol contains no more than 80*80% 1’s. In addition, the sine-cosine values are represented by integers in the range of 0 to 15. In extreme cases, 80*80% 1’s are required to multiply with these sine-cosine values. The sine-cosine values involved in the multiplication are considered as the mean value, 7.5, and then the multiplication result is 80*80% *7.5. The result of the current symbol needs to be added to that of the previous symbol, so the accumulated result can be 80*80% *7.5*2 = 960. To simplify calculating the square of large number, the *num* in Eq. (18) is set to 16. Thus, 80*80% *7.5*2/16 = 960/16 = 60. The division by 16 yields the same result as a 4-bit right shift. In order to prevent some accumulated results divided by 16 from exceeding 60, we add an overflow control. If any results are greater than 60, the results are set to 60.

In Algorithm 1 , the slow decay and fast decay factors are set to 1/2 and 4, and the µis 0.06, and then we get the segmented line equation

(21) \begin{eqnarray*}\begin{array}{@{}l@{}} \displaystyle \hat {f}(x)= \left\{ \begin{array}{@{}l@{}} \displaystyle 2x,s{p}_{0}\leq x\leq s{p}_{1}\\ \displaystyle 6x-8,s{p}_{1}< x\leq s{p}_{2}\\ \displaystyle 11x-28,s{p}_{2}< x\leq s{p}_{3}\\ \displaystyle 18x-77,s{p}_{3}< x\leq s{p}_{4}\\ \displaystyle 26x-165,s{p}_{4}< x\leq s{p}_{5}\\ \displaystyle 35x-300,s{p}_{5}< x\leq s{p}_{6}\\ \displaystyle 46x-520,s{p}_{6}< x\leq s{p}_{7}\\ \displaystyle 58x-832,s{p}_{7}< x\leq s{p}_{8}\\ \displaystyle 70x-1216,s{p}_{8}< x\leq s{p}_{9}\\ \displaystyle 83x-1710,s{p}_{9}< x\leq s{p}_{10}\\ \displaystyle 98x-2385,s{p}_{10}< x\leq s{p}_{11}\\ \displaystyle 113x-3180,s{p}_{11}< x\leq s{p}_{12} \end{array} \right. \end{array}\end{eqnarray*}

where the values of *sp*_0_, *sp*_1_, *sp*_2_, ..., *sp*_12_ are 0, 2, 4, 7, 11, 15, 20, 26, 32, 38, 45, 53, 60. We can explore other appropriate values in conjunction with optimization algorithms such as particle swarms (Chen et al., 2022). By replacing the multiplication in Eq. (21) with a shift operation, Eq. (21) become

(22) \begin{eqnarray*}\begin{array}{@{}l@{}} \displaystyle \hat {f}(x)= \left\{ \begin{array}{@{}l@{}} \displaystyle x< < 1,s{p}_{0}\leq x\leq s{p}_{1}\\ \displaystyle x< < 2+x< < 1-8,s{p}_{1}< x\leq s{p}_{2}\\ \displaystyle x< < 3+x< < 1+x-28,s{p}_{2}< x\leq s{p}_{3}\\ \displaystyle x< < 4+x< < 1-77,s{p}_{3}< x\leq s{p}_{4}\\ \displaystyle x< < 4+x< < 3+x< < 1-165,s{p}_{4}< x\leq s{p}_{5}\\ \displaystyle x< < 5+x< < 1+x-300,s{p}_{5}< x\leq s{p}_{6}\\ \displaystyle x< < 5+x< < 3+x< < 2+x-520,s{p}_{6}< x\leq s{p}_{7}\\ \displaystyle x< < 5+x< < 4+x< < 3+x-832,s{p}_{7}< x\leq s{p}_{8}\\ \displaystyle x< < 6+x< < 2+x< < 1-1216,s{p}_{8}< x\leq s{p}_{9}\\ \displaystyle x< < 6+x< < 4+x< < 1+x-1710,s{p}_{9}< x\leq s{p}_{10}\\ \displaystyle x< < 6+x< < 5+x< < 1-2385,s{p}_{10}< x\leq s{p}_{11}\\ \displaystyle x< < 6+x< < 5+x< < 4+x-3180,s{p}_{11}< x\leq s{p}_{12} \end{array} \right. \end{array}\end{eqnarray*}

where the < < indicates that the variable *x* is left-shifted.

**Figure 3:**
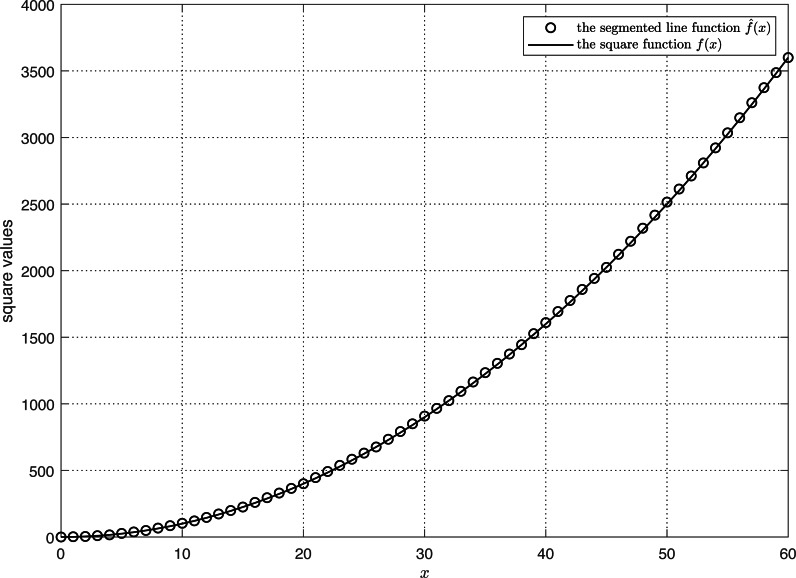
The comparison of segmented line prediction and actual square.

As shown in Fig. 3, we compare the segmented line function $\hat {f}(x)$ with the square function *f*(*x*). It is seen that the two curves exhibit a high degree of concordance, suggesting a strong resemblance between them. Using Eq. (23), the correlation coefficient *rela* between the estimated and actual square values equals 1.

(23) \begin{eqnarray*}\begin{array}{@{}l@{}} \displaystyle rela= \frac{\sum _{i=1}^{n} \left\{ f({x}_{i})-E \left[ f({x}_{i}) \right] \right\} \left\{ \hat {f}({x}_{i})-E \left[ \hat {f}({x}_{i}) \right] \right\} }{\sqrt{\sum _{i=1}^{n}{ \left\{ f({x}_{i})-E \left[ f({x}_{i}) \right] \right\} }^{2}\sum _{i=1}^{n}{ \left\{ \hat {f}({x}_{i})-E \left[ \hat {f}({x}_{i}) \right] \right\} }^{2}}} \end{array}\end{eqnarray*}

where *E*[*f*(*x*_*i*_)] denotes the expected actual value and $E \left[ \hat {f}({x}_{i}) \right] $ denotes the expected estimated value.

(24) \begin{eqnarray*}\begin{array}{@{}l@{}} \displaystyle td= \frac{rela}{\sqrt{ \frac{1-rel{a}^{2}}{n-2} }} \end{array}.\end{eqnarray*}

Then, we define a variable *td* according to Eq. (24), consult the *t*-distribution table, and obtain a *p*-value of 0 that is less than the significance level (*p* = 0.05). As a result, the correlation coefficient *rela* is regarded as significant. The $\hat {f}(x)$ and *f*(*x*) are completely correlated.

(25) \begin{eqnarray*}\begin{array}{@{}l@{}} \displaystyle {R}^{2}=1- \frac{\sum _{i=1}^{n}{ \left[ \hat {f}({x}_{i})-f({x}_{i}) \right] }^{2}}{\sum _{i=1}^{n}{ \left[ \bar {f}({x}_{i})-f({x}_{i}) \right] }^{2}} . \end{array}\end{eqnarray*}

Next, we examine the *R*^2^ relationship between $\hat {f}(x)$ and *f*(*x*) as stated in Eq. (25). The calculated value of *R*^2^ is zero, demonstrating that the variance of the difference between $\hat {f}(x)$ and *f*(*x*) is 0% of the variance of *f*(*x*). The variance of the difference between $\hat {f}(x)$ and *f*(*x*) is extremely small, indicating that $\hat {f}(x)$ and *f*(*x*) are quite close in value.

### Implementation result

We use a Spartan6 XC6SLX25 to implement the FPGA. Table 1 illustrates the resources consumed by spectrum shaping encoder based on MASP-R and MASP-NRS. These two MASPs employ the same decoding technique, and the hardware resources of decoder are detailed in Table 2. We can see that the encoder consumes more resources than the decoder, since the former one implements the MASP-R/MASP-NRS algorithms. The encoder consumes more 1,500 slice registers than the decoder. Also, it consumes twice as many LUT slices as decoder, due to MASP-R/MASP-NRS needs combinatorial logics such as addition. In particular, the encoder with MASP-R employs two DSPs to calculate the remainder and square operation as in Eq. (7), whereas the encoder with MASP-NRS needs no DSP. Since MASP-NRS eliminates the remainder operation, the corresponding encoder occupies some Block RAMs. Based on the Spartan6 XC6SLX25 implementation, the encoder and decoder with MASP-NRS can operate at frequencies of 121.560 MHz and 164.401 MHz, respectively.

**Table 1:** The hardware sources of spectrum shaping encoder based on MASP-R and MASP-NRS.

Logic utilization	Method	Used	Available	Utilization
Slice registers	MASP-R	2,966	30,064	9%
	MASP-NRS	1,965	30,064	6%
Slice LUTs	MASP-R	5,250	15,032	34%
	MASP-NRS	4,273	15,032	28%
Block RAM/FIFO	MASP-R	0	52	0%
	MASP-NRS	24	52	46%
BUFG/BUFGCTRLs	MASP-R	1	16	6%
	MASP-NRS	1	16	6%
DSP48E1s	MASP-R	2	38	5%
	MASP-NRS	0	38	0%

**Table 2:** The hardware sources of spectrum shaping decoder.

Logic utilization	Used	Available	Utilization
Slice registers	864	30,064	2%
Slice LUTs	1,686	15,032	11%
Block RAM/FIFO	0	52	0%
BUFG/BUFGCTRLs	1	16	6%
DSP48E1s	0	38	0%

**Figure 4:**
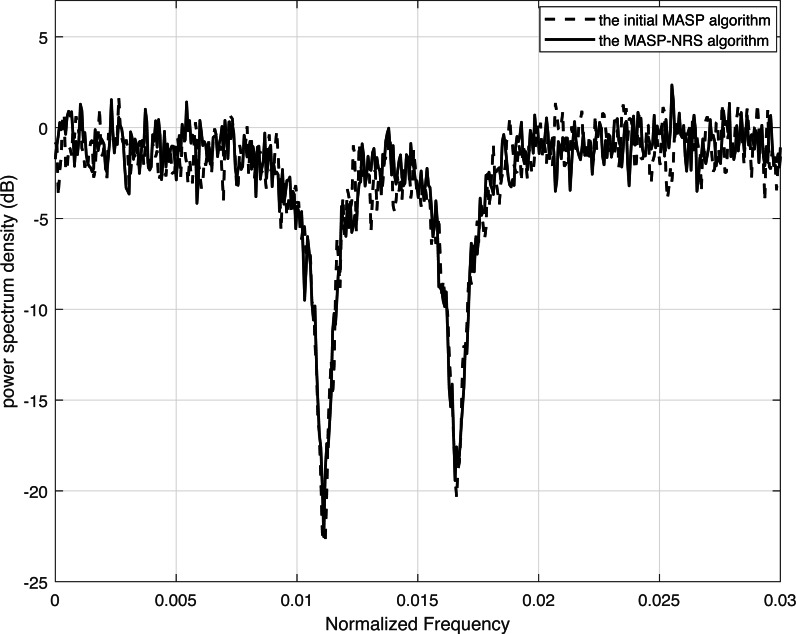
Comparison of the power spectrum density of MASP and MASP-NRS.

Figure 4 demonstrates the power spectrum densities for the same spectrum code. The dashed curve corresponds to the result of the initial MASP which is depicted in Eq. (7), while the solid curve represents the result of the MASP-NRS. Both curves use a code length of 80 bits, and the encoding and decoding methods are similar, except for the difference in the accumulated signal power method and scrambling. In Fig. 4, we can see that the MASP can generate spectrum nulls of −22.8 dB at frequency 1/90 and −20.0 dB at frequency 1/60. The improved algorithm MASP-NRS obtains spectrum nulls of −22.5 dB and −19.4 dB at frequencies 1/90 and 1/60, respectively. The spectrum nulls of MASP-NRS are 98.7% and 97.0% of those of the MASP, with losses of 1.3% and 3% due to truncation operations in MASP-NRS.

## Discussion and conclusion

In this research, we enhance the encoder–decoder algorithms for spectrum shaping codes in order to facilitate hardware implementation. We improve the scrambling algorithm and provide a mathematical description of the *k*-constrained algorithm. Concerning both descrambling and scrambling, we employ parallel operations that can be executed within a single schedule. We propose an enhanced MASP-R algorithm to compute remainder operations for sine-cosine accumulation; however, its execution in parallel is challenging due to its significant time complexity. Thus, we further present a MASP-NRS algorithm that quantizes sine-cosine values with short bit-width and stores them in ROM, eliminating the remainder operation. In particular, the MASP-NRS allows parallel operations for the sine-cosine accumulation within a single clock. It is capable of resolving the parallelization issue that plagued the initial MASP. Furthermore, we put forward a search algorithm that utilizes two approaches: dynamical error and balanced-coefficient mean square error. The search algorithm generates a curve $\hat {f}(x)$ similar to the square function *f*(*x*). By employing correlation and *R*^2^ analysis, it is possible to ascertain that *f*(*x*) and $\hat {f}(x)$ are almost equivalent. The complexity is reduced by two orders of magnitude through substituting the square operation in MASP with $\hat {f}(x)$. Finally, the encoder–decoder of shaping codes is executed utilizing the Spartan6 XC6SLX25. The synthesis results show that the decoder is simpler than the encoder since it does not have to calculate the accumulated signal power. Furthermore, we demonstrate that the performance of initial MASP and MASP-NRS is nearly identical, yielding spectrum nulls of approximately −22.8 dB, which confirms the accuracy of the proposed algorithm.

## Supplemental Information

10.7717/peerj-cs.1883/supp-1 Supplemental Information 1 Initial input data

10.7717/peerj-cs.1883/supp-2 Supplemental Information 2 The code to generate the result of the article

10.7717/peerj-cs.1883/supp-3 Supplemental Information 3 The code to produce Fig. 4

10.7717/peerj-cs.1883/supp-4 Supplemental Information 4 The key code that used in MASP-NRS

## Additional Information and Declarations

### Data Availability

The following information was supplied regarding data availability: The code is available in the Supplemental File.
